# Resveratrol Attenuates Diabetic Nephropathy via Modulating Angiogenesis

**DOI:** 10.1371/journal.pone.0082336

**Published:** 2013-12-03

**Authors:** Donghai Wen, Xinzhong Huang, Min Zhang, Liying Zhang, Jing Chen, Yong Gu, Chuan-Ming Hao

**Affiliations:** Department of Internal Medicine, Huashan Hospital, Fudan University, Shanghai, China; Fondazione IRCCS Ospedale Maggiore Policlinico & Fondazione D’Amico per la Ricerca sulle Malattie Renali, Italy

## Abstract

Angiogenesis plays an important role in the pathogenesis of diabetic nephropathy (DN). In the present study, we investigated the therapeutic potential of resveratrol, a polyphenol with antiangiogenic activity in DN. In a type 1 diabetic rat model, resveratrol treatment blunted the increases of urine albumin excretion, kidney weight and creatinine clearance rate. The increases of glomerular diameter, mesangium accumulation, glomerular basement membrane thickness and renal fibrosis in diabetic rats were also reduced by resveratrol treatment. In the diabetic kidney, increased expression of vascular endothelial growth factor (VEGF), Flk-1 and angiopoietin 2, and reduced expression of Tie-2 were observed. These changes in angiogenic hormones and associated receptors were attenuated by resveratrol treatment. No changes in angiopoietin 1 expression were detected among each group of rats. Resveratrol also significantly downregulated high glucose-induced VEGF and Flk-1 expressions in cultured mouse glomerular podocytes and endothelial cells, respectively. These effects were attenuated by knocking-down silent information regulator 1 (Sirt1) expression. In contrast, upregulation of Sirt1 in cultured endothelial cells reduced Flk-1 expression. Increased permeability and cellular junction disruption of cultured endothelial cells caused by VEGF were also inhibited by resveratrol pretreatment. Taken together, the present study demonstrated that resveratrol may attenuate DN via modulating angiogenesis.

## Introduction

Diabetic nephropathy (DN) is the leading cause of end-stage renal disease (ESRD) in the United States, and affects approximately 40% of diabetic patients [1]. DN is also associated with increased cardiovascular mortality [2]. Since it has brought heavy burden to both the patients and the government, the study of its prevention and treatment is one of the top priorities for both endocrinologists and nephrologists all over the world. Currently, the main treatments for DN are glycemic, lipid and blood pressure control, plus renin-angiotensin-aldosterone system (RAAS) blockade, such as angiotensin-converting enzyme (ACE) inhibitors, angiotensin II receptor blockers (ARBs) [3]. However, there are still a great number of DN patients progressing into ESRD, even after the aggressive uses of these treatments [3,4]. Thus, novel therapeutic approaches are required. The involvement of various factors such as hyperglycemia, angiotensin II, advanced glycation end products (AGEs), oxidative stress, transforming growth factor β (TGF-β), plasminogen activator inhibitor 1 (PAI-1), and connective tissue growth factor (CTGF) in DN has been reported [5].

Recently, there is a growing body of studies showing that angiogenesis may play an important role in the pathogenesis of DN [6]. Newly formed renal capillaries have been demonstrated in DN patients [7,8]. A potent stimulator of angiogenesis, vascular endothelial growth factor (VEGF) and its type 2 receptor Flk-1 have also been reported to be increased in DN animal models and patients, especially in the early stages [8,9]. VEGF has also been shown to promote endothelial cell proliferation, migration, and tube formation [10]. VEGF also induces vascular permeability, and this function is mainly mediated by Flk-1 [11,12]. Thus, it is suggested that VEGF may cause increased glomerular permeability and albuminuria in DN [13]. Furthermore, direct inhibition of VEGF or Flk-1 signaling (with neutralizing antibodies or receptor tyrosine kinase inhibitor) could also attenuate DN in both mouse and rat models [14-16]. In addition, other anti-angiogenic reagents, such as tumstatin;endostatin;angiostatin;2-(8-hydroxy-6-methoxy-1-oxo-1H-2-benzopyran-3-yl) propionic acid (NM-3); and vasohibin-1, have also been reported to have therapeutic potentials in DN animal models [17-22]. 

Angiopoietin 1 (Ang-1), by binding to its receptor Tie-2, stabilizes the attachment of endothelial cells and promotes the maturation of newly formed capillaries [23]. In contrast, as a natural antagonist of Ang-1, angiopoietin 2 (Ang-2) competitively inhibits the binding and interaction between Ang-1 and Tie-2, thus loosens the attachment of endothelial cells and synergizes VEGF to promote angiogenesis [24,25]. Ang-1, Ang-2 and Tie-2 have also been shown to play important roles in the maturation of renal blood vessels during kidney development [26]. Up-regulation of Ang-2, associated with decreased Tie-2 expression, has been reported in DN animal models, although the expression of Ang-1 was not altered [17,18,20]. 

Resveratrol is a natural polyphenol extracted from many plants [27]. It has also been shown to alleviate diabetic cardiac dysfunction [28]. Studies have shown that resveratrol potently suppresses VEGF expression and secretion, possibly through the inhibition of hypoxia-induced factor 1α (HIF-1α) [29-32]. Resveratrol also inhibits angiogenesis induced by VEGF, mainly through interruption of Src-dependent vascular endothelial cadherin tyrosine phosphorylation [33]. In addition, down-regulation of Flk-1 expression by resveratrol has also been demonstrated [34]. Furthermore, it has also been found that resveratrol has anti-angiogenic effect on tumor growth in vivo [35]. In addition, resveratrol is highly-orally absorbed and well tolerated by patients [36,37].

On the other hand, silent information regulator 1 (Sirt1) that can be activated by resveratrol, has been shown to have protective effects in diabetes and its complications [38]. As a class iii histone deacetylase, Sirt1 deacetylates many transcriptional factors, such as p53, FOXO, NF-κB, PGC-1α, LXR, and *etc* [39], exerting diverse cellular functions including cell fate determination, inflammatory response, energy metabolism, and environmental stress response [40]. We and others recently showed that Sirt1 activation could protect mouse kidney from oxidative stress [41,42]. Furthermore, Sirt1 has also been demonstrated to down-regulate VEGF expression both in vivo and in vitro, through deacetylating HIF-1α [43]. In the present study, we tested the hypothesis that resveratrol may also activate Sirt1, inhibiting VEGF signaling system and angiogenesis, and consequently attenuate DN.

## Methods

### Animal study

The principles of laboratory animal care were followed. The experimental protocol was approved by the Animal Study Committee of Huashan Hospital, Fudan University (Shanghai, China). A total of 60 Sprague-Dawley male rats weighing 180-200g were uninephrectomized from the right side. One week later, these rats were then randomly divided into three groups. The control group (CON, 10 rats) received once intraperitoneally injected 1ml citrate sodium buffer (CBS, pH 4.0), and larvaged with 5ml/kg body weight/day normal saline. The diabetic nephropathy group (DN, 25 rats) received once intraperitoneally injected streptozocin (STZ, 50mg/kg body weight), and larvaged with 5 ml/kg body weight/day normal saline. The treatment group (DN+RSV, 25 rats) received once intraperitoneally injected STZ (50mg/kg body weight), and larvaged with 20mg/kg body weight/day resveratrol (Copalyton Chemical Materials Co., Ltd, Shanghai, China) suspended in normal saline. Eight weeks later, there were 10 rats survived in each group. Urine samples were collected for measurement of albuminuria and creatinine before sacrificing the rats. All surgery was performed under sodium pentobarbital anesthesia, and all efforts were made to minimize suffering. Kidneys and blood samples were then obtained for subsequent analysis.

### Assessment of blood glucose and renal function

The levels of the blood glucose of the rats were determined by the blood glucose meter (Bayer Healthcare). To measure the urinary albumin excretion rate (UAER), the rats were housed in metabolic cages for 24h, and urine samples were collected for the assessment of albumin concentration by enzyme-linked immunosorbent assay (Orion Diagnostica Oy, Finland). Serum and urine creatinine concentrations were measured by an auto-analyzer. 

### Histolopathology and immunohistochemsitry analysis

The kidney samples were fixed with 4% paraformaldehyde for 4h, and embedded in paraffin. Sections (2μm thick) were stained with periodic acid Schiff (PAS) for light microscopic analysis. For immunohistochemistry examination, kidney sections were immunostained using immunoperoxidase technique with Vector ABC kit (Vector laboratories, USA). Briefly, sections (3μm thick) were blocked with 3% bovine serum albumin for 30 minutes at room temperature, and incubated overnight at 4°C with the primary antibodies: 1) rabblit anti-collagen type iv antibody (Boster Bio-engineering Co., Ltd, Wuhan, China); 2) rabbit anti-transforming growth factor β1 (TGF-β1) antibody (Santa Cruz Biotechnology, USA), and 3) rabbit anti-Sirt1 antibody (Millipore, USA). Sections were then washed and incubated with biotinylated secondary antibodies for 60 minutes at room temperature. Biotin was identified and visualized with diaminobenzidine. Random thirty glomeruli from each renal cortical area were observed, and images were then analyzed with Image Pro Plus 6.0 edition (Media Cybernetics, USA) for the determination of glomerular diameter, mesangial accumulation, and immunostained area.

### Glomerular basement membrane thickness assessment

Kidney tissues were fixed with 3% glutaraldehyde and embedded in Epon. The specimens were thin-sectioned and examined under transmission electron microscope. Five to ten glomeruli per kidney were randomly taken at 10,000 magnification for each rats. Glomerular basement (GBM) thickness was assessed at three different sites of cross-sectioning, with the aid of Image Pro Plus.

### Normal human and mouse kidney tissues

Normal human kidney tissues were obtained from surgical nephrectomy because of renal tumors. The kidney tissues were obtained with written informed consent from the patients to be used for research purposes after the diagnostic workup was completed. The study's protocol was approved by the Fudan University Huashan Hospital Ethics Committee. These tissues were obtained from macroscopically normal portion of kidney located at some distance from the neoplasm. Normal mouse kidney tissues were obtained from 9 months old C57bl6 male mice. The experimental protocol was approved by the Animal Study Committee of Huashan Hospital, Fudan Univeristy (Shanghai, China). All surgery was performed under sodium pentobarbital anesthesia, and all efforts were made to minimize suffering. No pathological findings were observed in both these normal tissues.

### Immunofluorescence

Cultured glomerular endothelial cells were washed with PBS and fixed with cold acetone. After three more washes and penetration of the cell membrane with 0.3% Triton X-100, the fixed tissue or cell sections then were incubated overnight at 4°C with: 1) rabbit anti-ZO-1 antibody (Boster Bio-engineering); 2) rabbit anti-claudin-5 antibody (Abcam, USA). After washing, the cells were then incubated with Cy3 or Cy2-conjugated secondary antibody (Millipore) for 60 min at room temperature, and viewed with immunofluorescence microscopy.

### Cells culture

Conditionally immortalized mouse podocytes were provided by Dr. Mundel [44], and cultured as previously described [45]. Briefly, cells were cultivated with RPMI 1640 (Gibco) containing 10% fetal bovine serum (FBS, Gibco) at 33°C to propagate. To induce differentiation, podocytes were maintained at 37°C for 14 days. Differentiated podocytes were used in this experiment. For endothelial cell culture, an immortalized mouse cell line was used [46]. Endothelial cells were cultivated in RPMI 1640 media supplemented with 10% FBS at 37°C. Podocytes or endothelial cells were serum starved for 24h, and then exposed to media containing normal glucose plus mannitol (5.6mM+24.4mM, NG+M) or high glucose (30mM, HG) with or without resveratrol (Sigma, USA). Cells were harvested for either protein or RNA assay. Podocytes conditioned cultured media were collected for the measurement of VEGF concentration.

### Modulation of Sirt1 expression in cultured cells

Lentivirus carrying selective Sirt1 shRNA was used to downregulate Sirt1 expression in cultured podocytes or endothelial cells as we previously described [41]. Briefly, HEK293T cells were cotransfected with lentiviral pLKO.1 plasmid carrying scrambled shRNA or Sirt1-selective shRNA (Sigma, SHCLNG-NM_019812), psPAX2 packaging plasmid, and pMD2.G envelop plasmid using FuGENE (Roche). After transfection, culture media containing lentiviral particles were collected and then infected to the cultured podocytes or endothelial cells. To upregulate Sirt1 expression, pCruzHA empty vector or pCruzHA-Sirt1 plasmid was transfected into the cultured endothelial cells using lipofectamine 2000 (Invitrogen, USA) as previously described [47]. 72 hours after the infection or transfection, immunoblot was performed to examine the efficiency as described before [48].

### Western blot analysis

Western blot was performed as described previously [49]. In brief, protein extracts were separated by electrophoresis on 10% SDS-PAGE and transferred to nitrocellulose membranes, which were then washed and incubated with blocking buffer (5% nonfat milk in PBS containing 0.1% Tween 20) for 1h at room temperature. The membranes were then incubated overnight at 4°C with the primary antibodies: 1) rabbit anti-VEGF antibody (Santa Cruz Biotechnology); 2)rabbit anti-Flk-1 antibody (Santa Cruz Biotechnology); 3) rabbit anti Ang-1 antibody (Santa Cruz Biotechnology) ; 4) rabbit anti-Tie-2 antibody (Santa Cruz Biotechnology); 5)rabbit anti-fibronectin antibody (Boster Bio-engineering Co., Ltd, Wuhan, China); 6) rabbit anti-PAI-1 antibody (Santa Cruz Biotechnology); 7) rabbit anti-CTGF antibody (Boster Bio-engineering), and 8) mouse anti-β-actin antibody (Sigma). After 3 washes, the membranes were incubated with horseradishperoxidase-conjugated secondary antibodies (Santa Cruz Biotechnology) for 1h at room temperature followed by further washes. Antibody labeling was visualized via ECL (Amersham Biosciences, UK). 

### Quantitative real time reverse transcription polymerase chain reaction (qRT-PCR)

Total RNA was extracted from kidney tissues using TRIzol reagent (Invitrogen). Reverse transcription was performed using a first strand cDNA synthesis kit (Fermentas Life Sciences, USA). qRT-PCR was performed using a SYBR Green/ROX qPCR Master Mix kit under the manufacturer’s instruction protocol (Fermentas Life Sciences). The primers used were: Ang-2 (5’-GACCAGTGGGCATCGCTACG-3’, 5’-CATTGTCCGAATCCTTTGTGCT-3’); β-actin (5’-CACCCGCGAGTACAACCTTC-3’, 5’-CCCATACCCACCATCACACC-3’). Data was analyzed using the 2 (-Delta Delta C(T)) method.

### Enzyme-linked immunosorbent assay (ELISA)

VEGF concentration in the podocytes conditioned cultured media collected above was determined by a mouse VEGF ELISA kit (Boster Bio-engineering) under the manufacturer’s instruction protocol. VEGF-164 was measured in duplicate 100 microliters samples. 

### Endothelial cells permeability assay

In vitro vascular permeability assay kit (Millipore) was used to measure the glomerular endothelial cells permeability under the manufacturer’s instruction protocol. Briefly, endothelial cells were seeded into the plate until a monolayer was formed. Media were then carefully removed and the inserts were transferred to fresh plate wells. After starved for 24 hours, cells were pretreated with or without resveratrol (25μM) for another 24 hours, and then treated with or without recombinant human VEGF165 (50ng/ml, R&D system) for 3 hours. The inserts were then transferred to the permeability detection plate, FITC-dextran was added and incubated for 5 minutes at room temperature. Plate solution was then transferred to a 96-well plate and read using a fluorometer with a 485nm and 530nm filter set.

### Statistical analysis

Data were shown as mean ± SEM. Statistical analysis was performed using SPSS 12.0 software. Independent Student’s *t* test or ANOVA were used to determine the significant differences. P<0.05 was considered significant.

## Results

### Resveratrol attenuated pathological changes in DN

**Figure 1:**
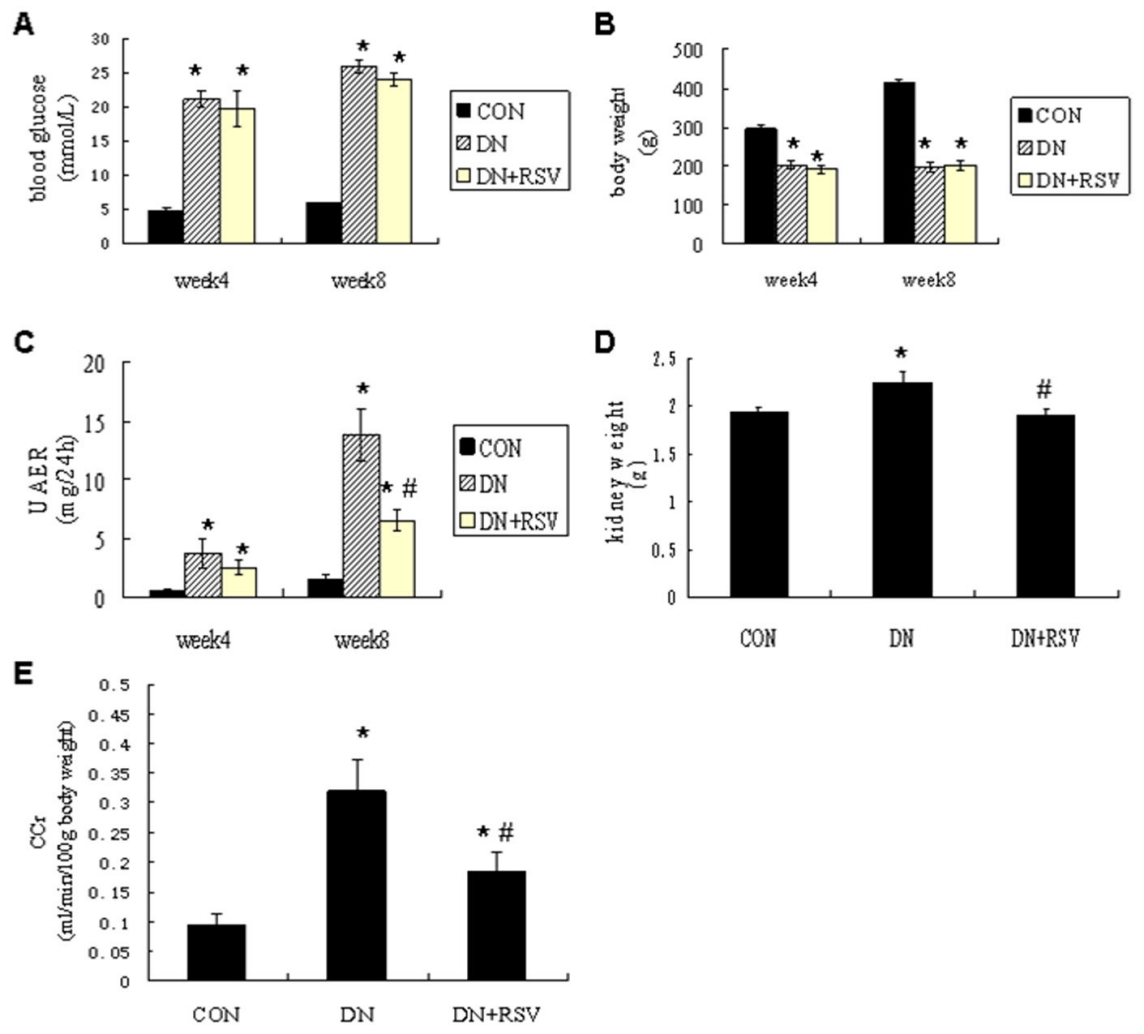
Effect of resveratrol on albuminuria and renal hyperfiltration in diabetic rats. The levels of blood glucose (A), body weight (B), albuminuria (C), kidney weight (D), and CCr (E) in each group of rats. *P<0.05 vs CON; #P<0.05 vs DN. CON, control; DN, diabetic nephropathy; RSV, resveratrol. UAER, urine albumin excretion rate; CCr, creatinine clearance rate.

**Figure 2:**
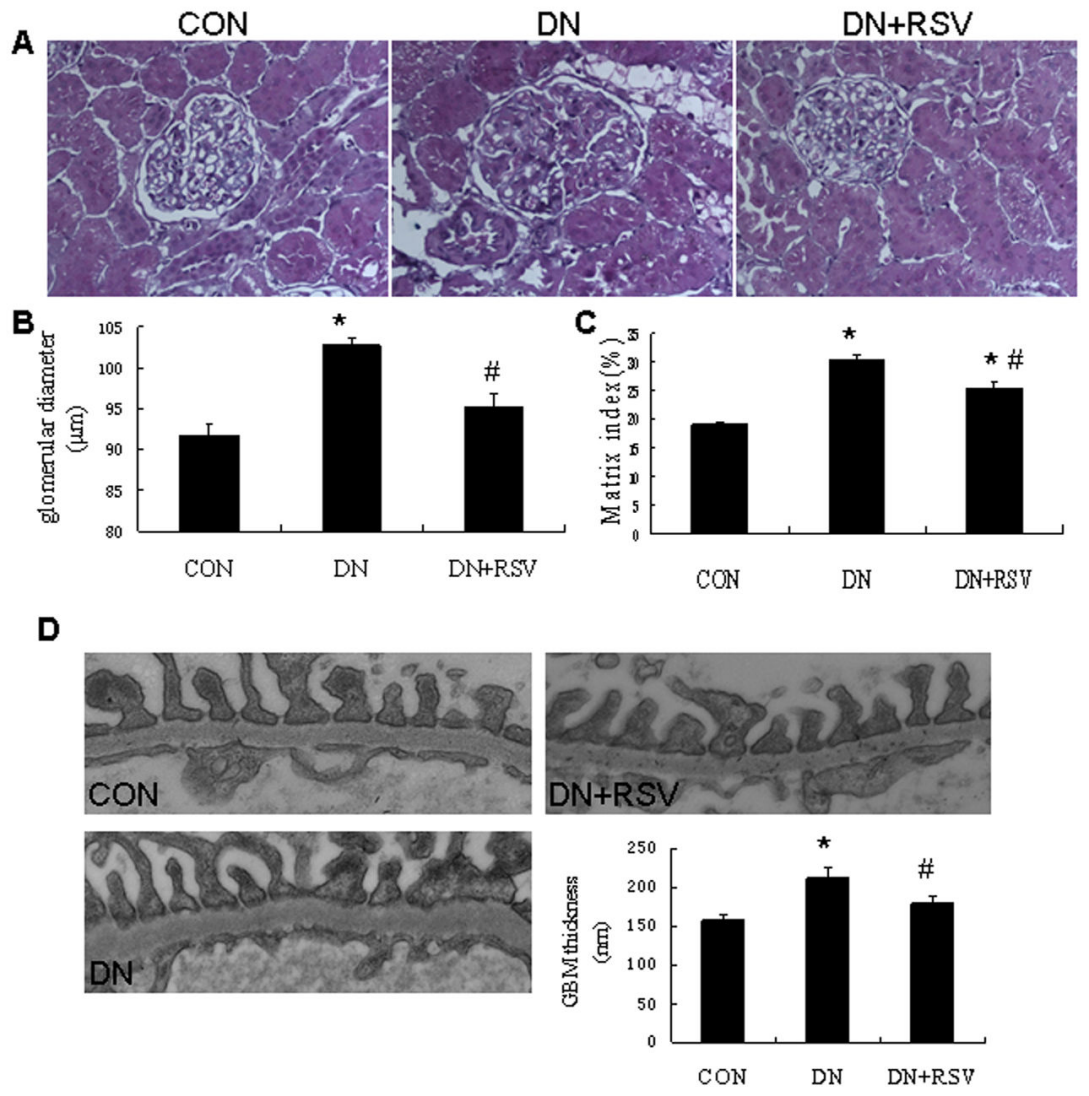
Effect of resveratrol on renal hypertrophy and GBM thickness in diabetic rats. (A) H&E staining of the kidney sections from each group of rats. (B,C) Glomerular diameter and matrix index of each group of rats. (D) GBM thickness analysis under transmission electronic microscopy (TEM) from each group of rats. *P<0.05 vs CON; #P<0.05 vs DN. CON, control; DN, diabetic nephropathy; RSV, resveratrol. GBM, glomerular basement membrane.

**Figure 3:**
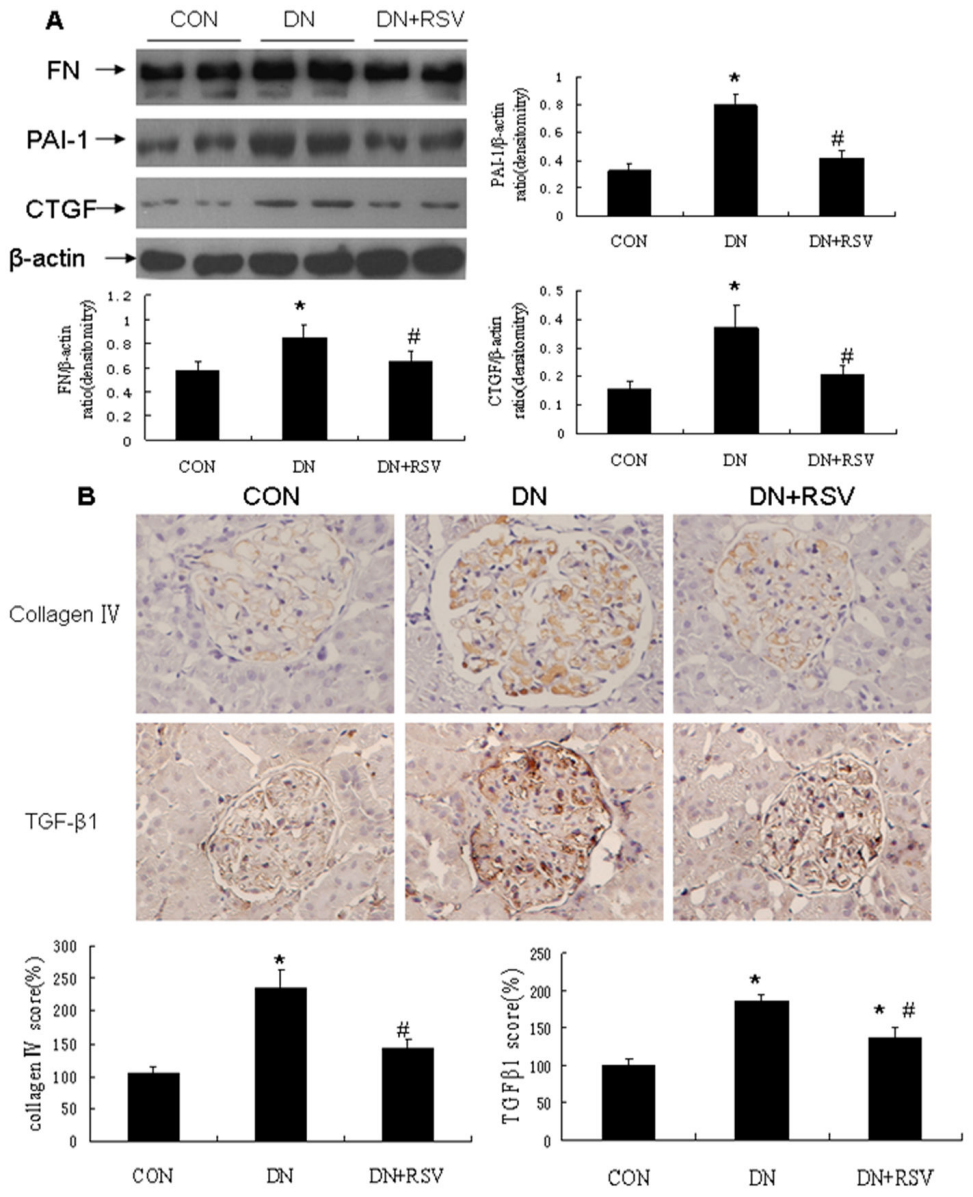
Effect of resveratrol renal fibrosis in diabetic rats. (A) Western blot analysis of FN, PAI-1, and CTGF in each group of rats. (B) Immunohistochemistry analysis of Collagen iv and TGF-β1 in each group of rats. *P<0.05 vs CON; #P<0.05 vs DN. CON, control; DN, diabetic nephropathy; RSV, resveratrol. FN, fibronectin; PAI-1, plaminogen activator inhibitor 1; CTGF, connective tissue growth factor; TGF-β1, transforming growth factor β1.

The blood glucose was significantly increased in the diabetic rats compared with control rats, while the body weight was markedly reduced. Treatment with resveratrol did not significantly change the blood glucose level and body weight in the diabetic rats (Figure 1.A and B). The urinary albumin excretion rate (UAER) was markedly elevated in the diabetic rats, while treatment with resveratrol significantly decreased the UAER after 8 weeks of treatment (Figure 1.C, CON 1.53±0.38mg/24h; DN 13.81±1.25mg/24h; DN+RSV 6.55±0.57mg/24h). To evaluate the effects of resveratrol on the hyperperfusion and hyperfiltration in DN, kidney weight and creatinine clearance (CCr) were examined. Resveratrol treatment resulted in the suppression of the diabetes-induced increases of kidney weight (Figure 1.D, CON 1.94±0.04g; DN 2.26±0.11g; DN+RSV 1.91±0.07g) and CCr (Figure 1.E, CON 0.09±0.02ml/min/100g body weight; DN 0.32±0.05ml/min/100g body weight; DN+RSV 0.18±0.03ml/min/100g body weight). Histological examination of the kidney sections revealed hypertrophy and expansion of the mesangial area in the diabetic rats. Treatment with resveratrol remarkably attenuated glomerular hypertrophy and mesangial matrix accumulation compared with vehicle treated diabetic rats (Figure 2.A and B). The transmission electron microscopy study showed that the glomerular basement membrane (GBM) thickness was significantly increased in the diabetic rats compared with control rats, while resveratrol treatment remarkably suppressed the diabetes-induced GBM thickness (Figure 2.D, CON 158.33±6.66nm; DN 211.67±14.05nm; DN+RSV 179.67±7.64nm). To further evaluate the anti-fibrosis effect of resveratrol in DN, the expression levels of fibronectin (FN), plasminogen activator inhibitor 1 (PAI-1) and connective tissue growth factor (CTGF) were examined by Western blot analysis, and the type iv collagen expression and transforming growth factor β1 (TGF-β1) were determined by immunohistochemistry. Diabetic rats exhibited increased expression levels of type iv collagen, FN, PAI-1, TGF-β1 and CTGF compared with control rats in the renal cortex. Treatment with resveratrol resulted in the suppression of their expression in the diabetic rats（Figure 3.A and B,).

### Resveratrol modulated angiogenesis in DN

**Figure 4:**
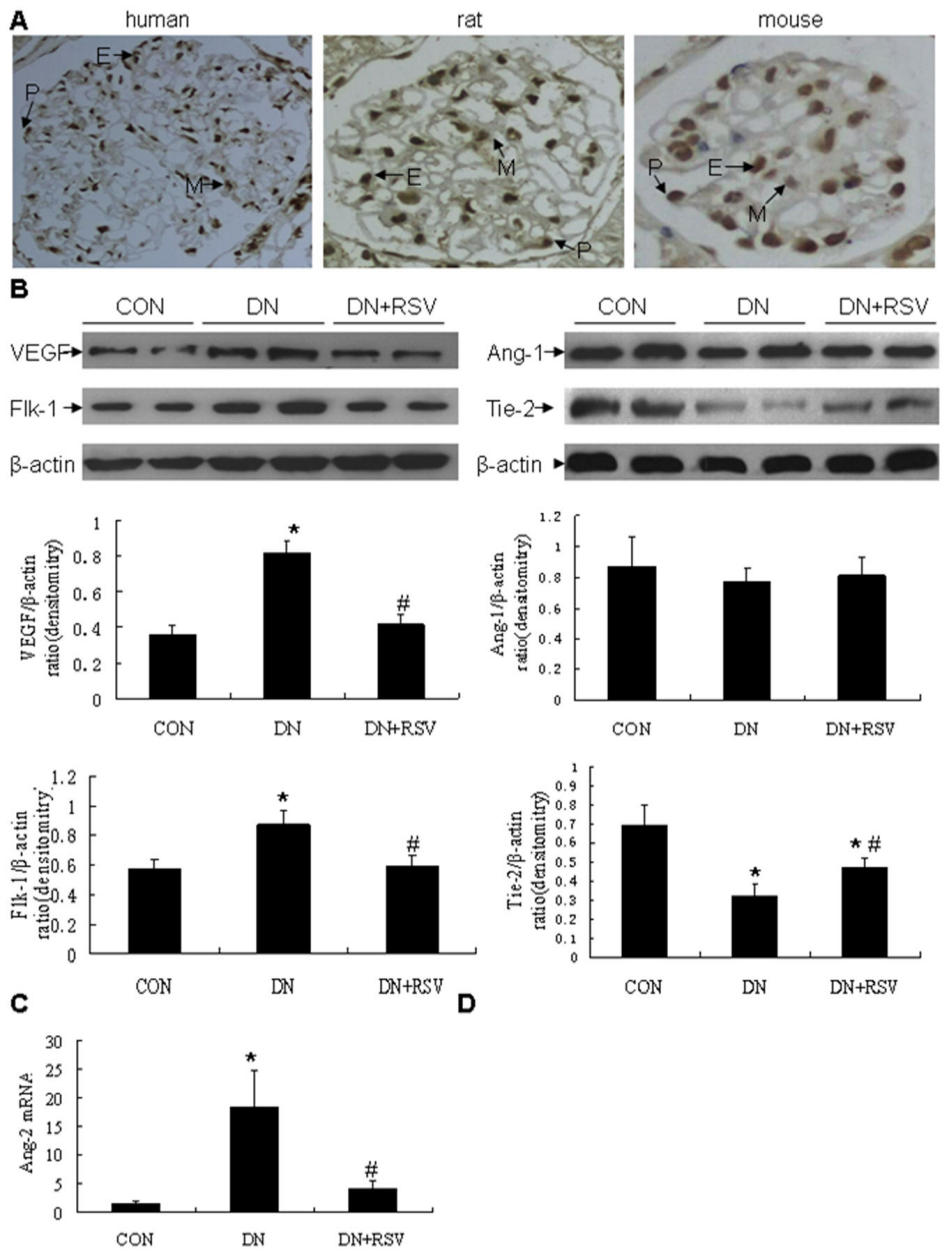
Effect of resveratrol on renal angiogenesis in diabetic rats. (A) Immunohistochemistry of Sirt1 expression in normal glomeruli of human, rat and mouse. (B) Western blot analysis of VEGF, Flk-1, Ang-1, and Tie-2 expression in the kidneys from each group of rats. (C) Realtime PCR analysis of renal Ang-2 mRNA transcription in each group of rats. *P<0.05 vs CON; #P<0.05 vs DN. P, podocyte; M, mesangial cell; E, endothelial cells; CON, control; DN, diabetic nephropathy; RSV, resveratrol.

Sirt1 expression was detected in healthy adult kidneys of human, mouse and rat, using immunohistochemistry with a Sirt1-specific antibody. Glomeruli from all three species showed Sirt1 staining in podocytes, endothelial cells and mesangial cells (Figure 4.A). The effect of resveratrol on the expression levels of VEGF, Flk-1, Ang-1 and Tie-2 in the renal cortex were then examined by Western blot analysis. Treatment with resveratrol remarkably suppressed the increases of VEGF and Flk-1 in the diabetic rats, while reduced the diabetes-induced decreased Tie-2 expression. The renal expression of Ang-1 was not different among each group of rats (Figure 4.B). Resveratrol treatment also significantly inhibited the increased transcription level of Ang-2 mRNA in the diabetic kidneys, as determined by real time RT-PCR (Figure 4.C).

### Resveratrol suppressed VEGF expression and secretion in cultured podocytes

**Figure 5:**
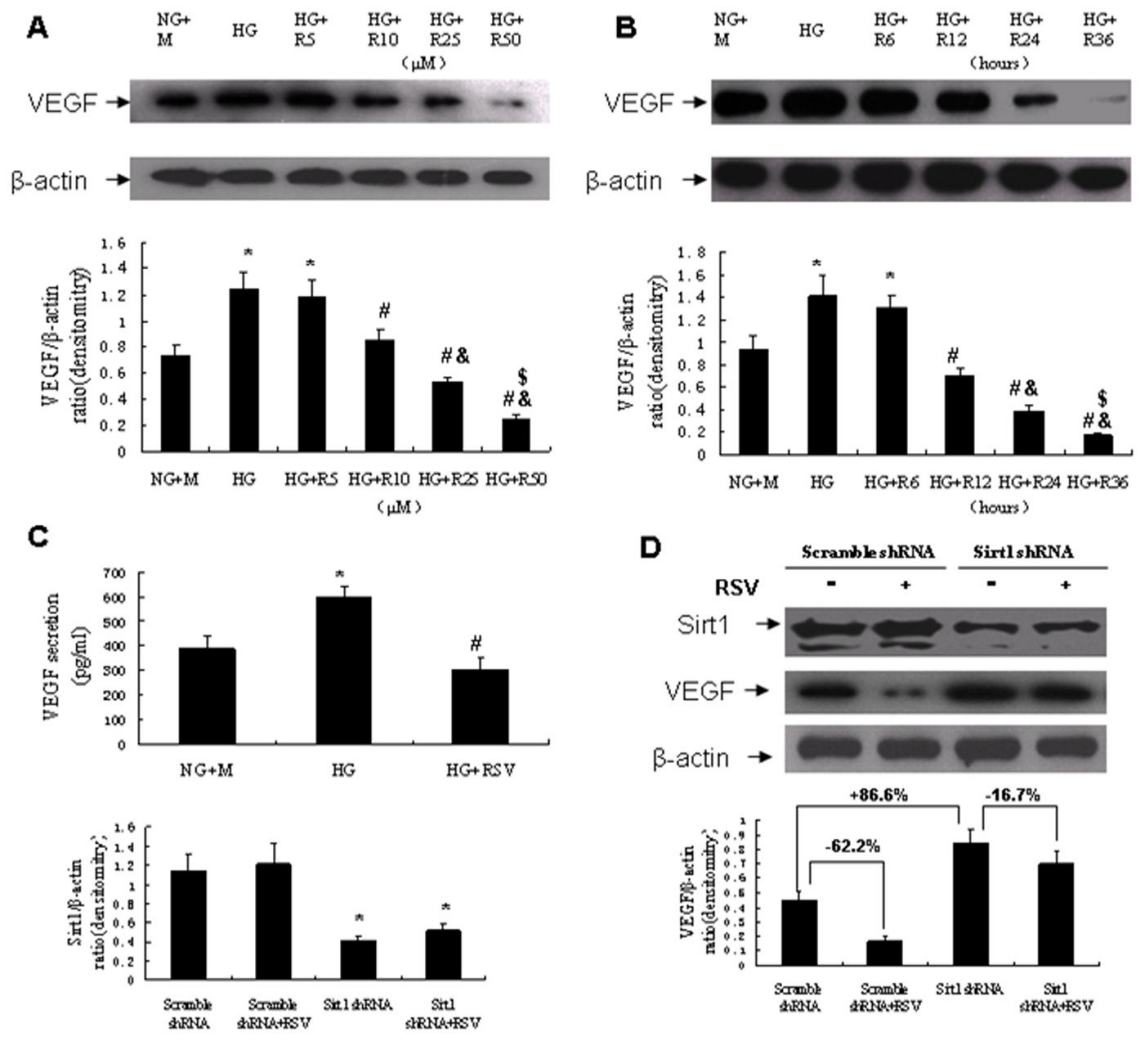
Effect of resveratrol on VEGF expression in cultured mouse podocytes. (A) Western blot analysis of VEGF expression in cultured mouse podocytes treated with resveratrol in various concentrations for 24 hours. *P<0.05 vs NG+M; #P<0.05 vs HG. &*P*<0.05 vs HG+R5; $*P*<0.05 vs HG+R10. (B) Western blot analysis of VEGF expression in cultured mouse podocytes treated with resveratrol (25μM) for various time periods. *P<0.05 vs NG+M; #P<0.05 vs HG. &*P*<0.05 vs HG+R12; $*P*<0.05 vs HG+R24. (C) ELISA analysis of VEGF concentrations in the cultured podocytes media treated with or without resveratrol (25μM) for 24 hours. *P<0.05 vs NG+M; #P<0.05 vs HG. (D) Western blot analysis of Sirt1 and VEGF expression in the lentivirus-infected cultured podocytes treated with or without resveratrol (25μM) for 24 hours. *P<0.05 vs Scramble shRNA. NG+M, normal glucose plus mannitol; HG, high glucose; R, RSV, resveratrol.

Mouse podocytes were cultured with normal glucose plus mannitol (NG+M) or high glucose (HG) with or without resveratrol. The VEGF expression level was determined by Western blot analysis. As compared with NG+M, HG significantly increased podocytes VEGF expression. Resveratrol decreased the HG-induced VEGF expression in a dose- and time-dependent manner (Figure 5). ELISA analysis showed that treatment with resveratrol (25μM) reduced HG-induced VEGF secretion in the cultured podocytes media (Figure 5.C). To examined whether Sirt1 is involved in the resveratrol-induced VEGF downregulation, podocytes Sirt1 expression was knocked down using a lentivirus containing Sirt1-specific shRNA, and cells were treated with or without resveratrol (25μM) for 24 hours after the infection. Sirt1 and VEGF expression levels were then determined by Western blot analysis. As compared with scramble shRNA, Sirt1 shRNA containing virus significantly down-regulated Sirt1 expression in cultured podocytes. In the podocytes infected with scramble shRNA lentivirus, resveratrol significantly down-regulated VEGF expression by 62.2%. However, in the podocytes infected with Sirt1 shRNA lentivirus, resveratrol reduced VEGF expression only by 16.7%. And the VEGF expression was markedly increased to 186.6% in the podocytes infected with Sirt1 shRNA lentivirus as compared with those infected with scramble shRNA lentivirus (Figure 5.D).

### Resveratrol suppressed Flk-1 expression in cultured glomerular endothelial cells

**Figure 6:**
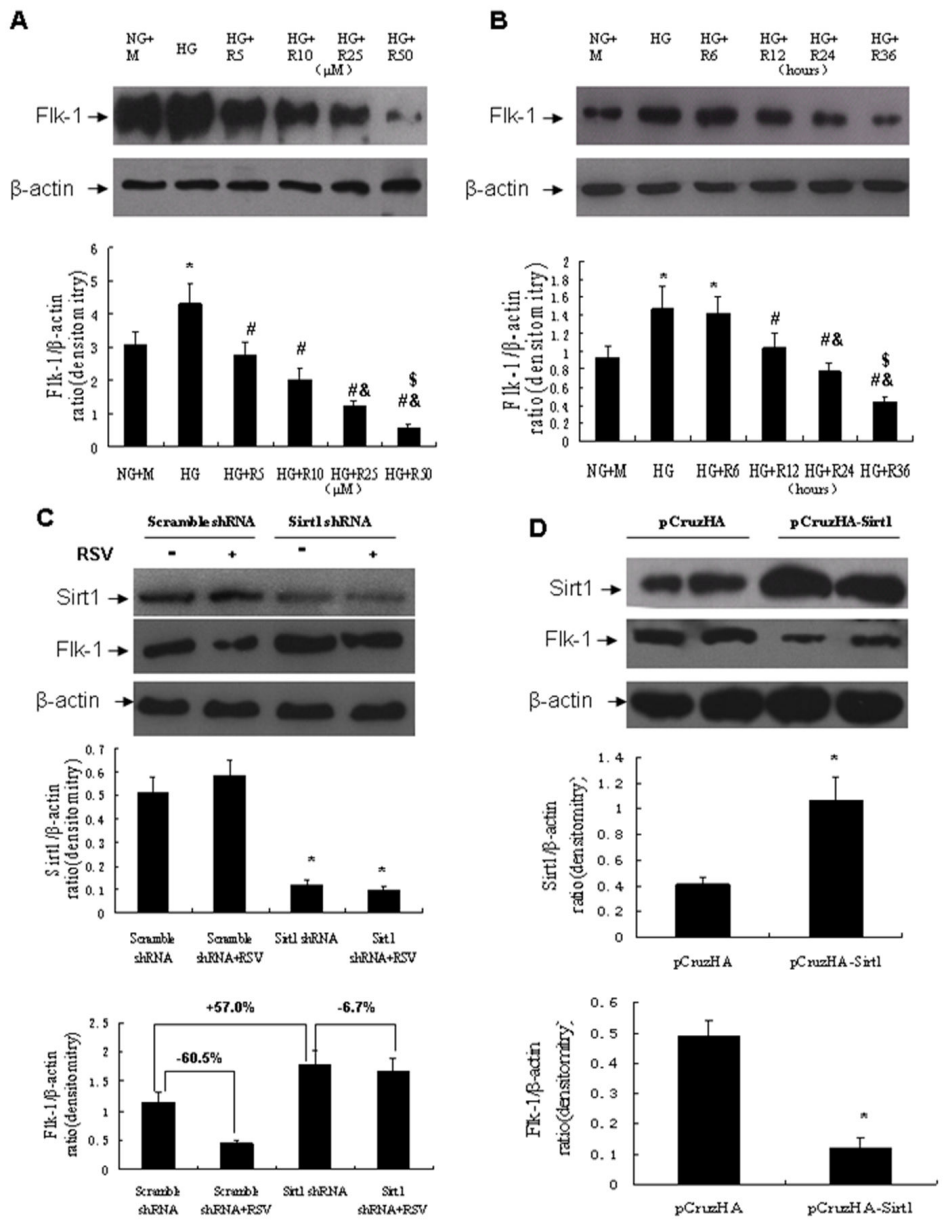
Effect of resveratrol on Flk-1 expression in cultured mouse glomerular endothelial cells. (A) Western blot analysis of Flk-1 expression in cultured mouse glomerular endothelial cells treated with resveratrol in various concentrations for 24 hours. *P<0.05 vs NG+M; #P<0.05 vs HG. &*P*<0.05 vs HG+R5; $*P*<0.05 vs HG+R10. (B) Western blot analysis of Flk-1 expression in cultured mouse glomerular endothelial cells treated with resveratrol (25μM) for various time periods. *P<0.05 vs NG+M; #P<0.05 vs HG. &*P*<0.05 vs HG+R12; $*P*<0.05 vs HG+R24. (C) Western blot analysis of Sirt1 and Flk-1 expression in the lentivirus-infected cultured mouse glomerular endothelial cells treated with or without resveratrol (25μM) for 24 hours. *P<0.05 vs Scramble shRNA. (D) Western blot analysis of Sirt1 and Flk-1 expression in the plasmids-transfected cultured glomerular endothelial cells. *P<0.05 vs pCruzHA. NG+M, normal glucose plus mannitol; HG, high glucose; R, RSV, resveratrol.

Mouse glomerular endothelial cells were incubated with normal glucose plus mannitol (NG+M) or high glucose (HG) with or without resveratrol, Flk-1 expression level was determined by Western blot analysis. Endothelial cells incubated with HG had higher Flk-1 expression than those cultured with NG+M. Resveratrol dose- and time-dependently down-regulated the HG-induced Flk-1 expression (Figure 6). To examined whether Sirt1 mediated the resveratrol-induced Flk-1, Sirt1 was knocked down in cultured endothelial cells using a lentivirus containing Sirt1-specific shRNA, and cells were then treated with resveratrol (25μM) for 24 hours after the infection. In the scramble shRNA infected cells, resveratrol markedly reduced Flk-1 expression by 60.5%. But in the Sirt1 shRNA infected cells, resveratrol decreased Flk-1 expression by a much less degree (6.7%). The Flk-1 expression of the Sirt1 shRNA infected cells was also 57.0% higher than those of the scramble shRNA infected cells (Figure 6.C). To further confirm the effect of Sirt1 on Flk-1 expression, Sirt1 was over-expressed using a pCruzHA-Sirt1 plasmid. As compared with cells transfected with pCruzHA control plasmid, in those transfected with pCruzHA-Sirt1, the Flk-1 expression was markedly decreased (Figure 6.D).

### Resveratrol ameliorated VEGF-induced hyperpermeability and cellular junction disruption in cultured glomerular endothelial cells

**Figure 7:**
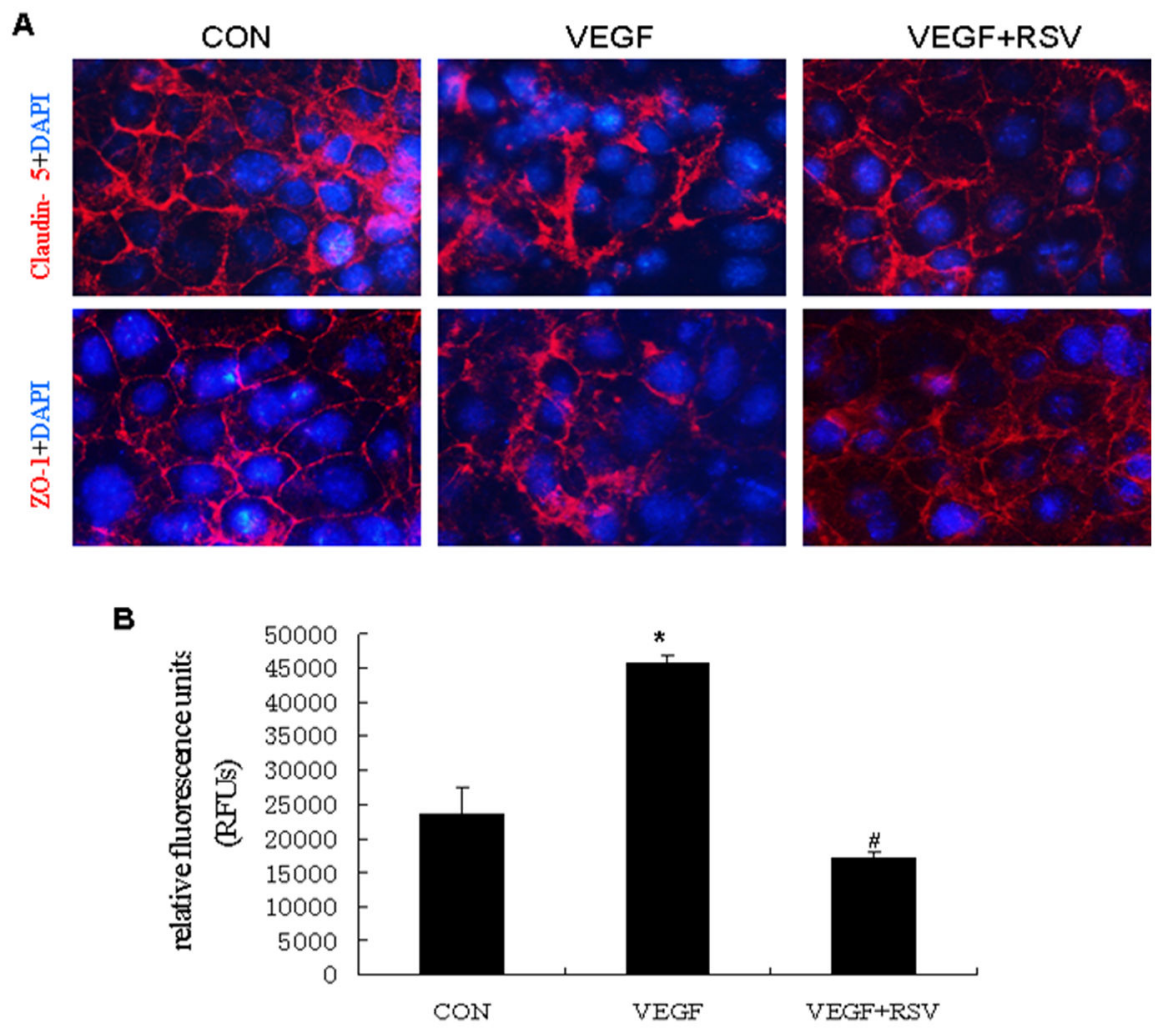
Effect of resveratrol on VEGF-induced cellular junction disruption and hyperpermeability in cultured mouse glomerular endothelial cells. Cultured mouse glomerular endothelial cells were pre-incubated with or without resveratrol (25μM) for 24 hours, and then treated with or without VEGF (50ng/mL) for 3 hours. (A) Immunofluorescence study of Claudin-5 and ZO-1 in each group of cells. (B) In vitro vascular permeability assay in each group of cells. *P<0.05 vs CON; #P<0.05 vs VEGF. CON. control; RSV, resveratrol.

In the cultured glomerular endothelial cells, treatment with VEGF (50ng/ml) for 3 hours caused cellular junction disruption as determined by zona occluden 1 (ZO-1) and claudin-5 immunofluorescence. Pretreatment with resveratrol (25μM) for 24 hours significantly alleviated this VEGF-induced cellular junction disruption (Figure 7.A). In the in vitro vascular permeability assay, VEGF also caused increased permeability of cultured glomerular endothelial cells, which was reduced by pretreatment with resveratrol (Figure 7.B).

## Discussion

Angiogenesis is composed of the following steps: 1) degradation of vascular basement membrane; 2) proliferation and migration of endothelial cells; 3) formation of endothelial tube; and 4) maturation of newly formed blood vessels [50]. VEGF-Flk-1 system induces the first two steps, while the latter two are mostly mediated by Ang-1-Tie-2 system. Ang-2, which is a natural antagonist of Ang-1, inhibits maturation of blood vessels by blocking Tie-2 signaling, and also induces angiogenesis in the presence of VEGF [24,25]. Newly formed blood vessels have high permeability because of the loosen attachment between endothelial cells, while maturation is associated with firm attachment, and thus results in non-leaky blood vessels [50]. It has been suggested that the abnormal angiogenesis in DN, which is mostly mediated by VEGF-Flk-1 system, is associated with increased permeability in the glomerular endothelial cells and leakage of plasma albumin into the urine, which further results in albuminuria and development and progression of DN [6]. The involvement of VEGF-Flk-1 signaling in DN has been demonstrated by a growing body of studies, suggesting the therapeutic potential of the inhibition of VEGF-Flk-1 signaling [16-22]. On the other hand, the maturation of newly formed blood vessels, which is mediated by Ang-1-Tie2 system, is associated with decreased permeability and leakage of plasma albumin. The therapeutic potential of Ang-1 has been demonstrated in diabetic retinopathy [51]. The present study identified Sirt1 as an important regulator of VEGF-Flk-1 signaling system and a potential therapeutic target for DN. 

In the present study, we demonstrated for the first time that resveratrol could attenuate DN via inhibiting the VEGF-Flk-1 system. Treatment with resveratrol significantly suppressed the expression levels of VEGF and Flk-1 in the diabetic rat kidneys. Further in vitro studies also showed that resveratrol inhibited both VEGF and Flk-1 expression in the cultured glomerular podocytes and endothelial cells, respectively, which were dependent, at least in part, on Sirt1. Treatment with resveratrol remarkably inhibited the VEGF-induced hyperpermeability in cultured glomerular endothelial cells, and attenuated the albuminuria, glomerular capillary density and other pathological changes in the diabetic kidneys. 

In addition, treatment with resveratrol also suppressed the increase of Ang-2 and attenuated the decrease of Tie-2 expression in the diabetic kidneys. Thus, we postulated that, through activating the Ang-1-Tie-2 signaling system, resveratrol might also promote the firm attachment between glomerular endothelial cells, which further decrease the leakage of plasma albumin into the urine.

Resveratrol treatment did not affect blood glucose, body weight and food consumption in the diabetic rats, suggesting that the beneficial effect of resveratrol on DN is independent of the blood glucose levels. 

The detailed mechanism by which Sirt1 inhibits VEGF and Flk-1 expression in the glomerular podocytes and endothelial cells is not well understood. Previous study demonstrated that Sirt1 could suppress the VEGF expression via deacetylating HIF-1α [43]. On the other hand, Flk-1 expression has been shown to be regulated by nuclear transcription factor κB (NF-κB), which can also be deacetylated by Sirt1 [52,53]. Several other signaling pathways such as Akt/FOXO3 pathways might also be involved in the treatment of DN by resveratrol [54]. This needs to be further explored.

In conclusion, we demonstrated here that resveratrol could attenuate DN via modulating the angiogenic factors. Given that resveratrol has high oral bioavailability and excellent safety profile in human studies and clinical trials [36,37], our studies convincingly indicate that resveratrol may be a potential treatment approach in DN patients.
